# Species-specific effect of macrobenthic assemblages on meiobenthos and nematode community structure in shallow sandy sediments

**DOI:** 10.1007/s00227-013-2329-y

**Published:** 2013-10-02

**Authors:** Barbara Urban-Malinga, Aleksander Drgas, Sławomira Gromisz, Natalie Barnes

**Affiliations:** 1 Department of Fisheries Oceanography and Marine Ecology, National Marine Fisheries Research Institute, 81-332 Gdynia, Poland; 2 Zoology Department, The Natural History Museum, Cromwell Road, London, SW7 5BD UK

## Abstract

Three functionally different macrofaunal species (the filter- and/or surface deposit-feeding polychaete *Hediste diversicolor*, and the suspension-feeding bivalves *Mya arenaria* and *Cerastoderma glaucum*) were introduced as single- and two-species treatments into microcosms containing sandy sediment with a natural meiofaunal community. *H. diversicolor* is a burrowing species building a system of galleries, *C. glaucum* lives actively near the sediment surface acting as a biodiffuser and *M. arenaria* buries deeply and leads a sessile lifestyle. It is shown that *H. diversicolor* extended the vertical distribution of meiofauna into deeper sediment layers compared to the control and non-*Hediste* treatments. The response of the nematode community varied significantly among treatments and was dependant on the macrobenthic species composition but not on the species number. Nematode assemblages in all treatments with the polychaete, both in monoculture and with *either* bivalve, differed significantly from those recorded in other treatments and were more similar than replicates within any other single treatment. *H. diversicolor* also appeared to have stimulated nematode species diversity. The present study demonstrated that the impact of macrobenthic assemblages on meiofauna is not a simple summation of individual species effects but is species specific.

## Introduction

Benthic macroinvertebrates may influence occurrence, composition and distribution of the meiobenthos directly through predation and indirectly through alterations in interstitial physico-chemical characteristics and processes rates (e.g. transport of organic matter, mineralization rates, distribution and exchange of solutes). Observations performed both in the field and/or under laboratory conditions provide evidence on a wide range of meiofaunal responses to the presence of different macro-organisms, particularly with regard to their different feeding modes and bioturbatory activities. These meiofaunal responses include changes in their density, vertical distribution, taxonomic composition, community structure and diversity (e.g. Reise 1979; Olafsson and Elmgren 1991; Olafsson et al. 1993; Warwick et al. 1997; Austen et al. 1998; Austen and Widdicombe 1998; Schratzberger and Warwick 1999a, b; Tita et al. 2000; Ullberg and Olafsson 2003; Braeckman et al. 2011a). According to recent findings, functionally contrasting macrobenthic species affect nematodes in different ways (Braeckman et al. 2011a). Our knowledge of the effects of macrofauna on meiofauna is still very limited, however, and it is difficult to apply any general assemblage theories and/or predict diversity or abundance patterns in meiofaunal assemblages as an effect of macrobenthic activity (see Olafsson 2003).

Although a considerable amount of published literature is dedicated to the study of the role of marine macrobenthos biodiversity, species identity and functional traits in structuring benthic ecosystem processes rates (Emmerson and Raffaelli 2000; Emmerson et al. 2001; Biles et al. 2003; Raffaelli et al. 2003; Mermillod-Blondin et al. 2005; Ieno et al. 2006), the knowledge of their effects on meiobenthos is still very limited (Austen et al. 1998; Braeckman et al. 2011a, b). Meiofauna, and nematodes in particular, directly or indirectly influence many processes associated with energy flow, mineralization rates and recirculation of nutrients in marine benthic systems (Platt and Warwick 1980; Heip et al. 1982, 1985). It seems essential, therefore, to identify rules concerning meiofauna function to better understand functioning of the whole benthic system.

Research to date has tended to focus on the effects of single, locally important macrofaunal species (Olafsson and Elmgren 1991; Olafsson et al. 1993; Warwick et al. 1997; Austen et al. 1998; Austen and Widdicombe 1998; Schratzberger and Warwick 1999a, b; Tita et al. 2000; Ullberg and Olafsson, 2003; Braeckman et al. 2011a). Yet, in the natural environment species co-exist in a network of mutual interconnections and relationships, and the impact of single species on meiofaunal assemblages may presumably differ from the impact of the same species co-existing in a multi-species complex. Is the effect of multi-species macrofauna assemblages on meiofauna a simple summation of effects or is it more species specific? Whether higher macrobenthic diversity supports higher meiofaunal diversity also remains unknown. These questions are particularly relevant when studying the consequences of change, such as climate change, in marine benthic systems, including changes in species composition (e.g. as an effect of biological invasions), biodiversity loss and potential knock-on effects to essential ecosystem services.

It is well established that deposit-feeding and burrowing marine invertebrates modify sediment chemistry and physical structure and through these modifications affect transport rates in the sediment, facilitate the exchange of solutes between sediment porewaters and overlying water, influence the distribution of dissolved reactants and products, increase the effective surface area for such processes and may stimulate microbial activity (Kristensen and Hansen 1999; Christensen et al. 2000; Kristensen 2001; Kristensen and Kostka 2005 and refs therein; Karlson et al. 2007; Braeckman et al. 2010, 2011b). Recent evidence shows that the effects on sediment processes of multi-species macrobenthic assemblages, where those species use and modify the same sediment space, are not simply the summation of individual species effects (Waldbusser et al. 2004; Mermillod-Blondin et al. 2005). Mermillod-Blondin et al. (2005) demonstrated that vertical sediment gradients and characteristics of interstitial processes are mainly due to the most efficient bioturbator, which masks the effects of other disturbers. This indicates the predominance of species-specific functional traits in structuring sediment processes rates. It is well documented that meiofauna, particularly free-living nematodes, are sensitive to shifts in the interstitial environment, responding rapidly on both large and very small scales (Platt and Warwick 1980; Heip et al. 1982, 1985). Therefore, it can be hypothesized that the response of nematode communities to the presence of macrobenthic assemblages, which are composed of species characterized by various benthic activity types, is affected by the macrobenthic species composition and is species specific.

In this study, we have focused on the effect of three macrofaunal species typical for the shallow benthic environments of the southern Baltic coast, but differing considerably in terms of feeding activity and benthic lifestyle. We introduced these species as single- and two-species treatments into microcosms containing sediment with a natural meiofaunal community. We then examined the nematode community for structural changes in response to the presence of various macrobenthic assemblages.

## Materials and methods

### Characteristics of the study site

**Fig. 1:**
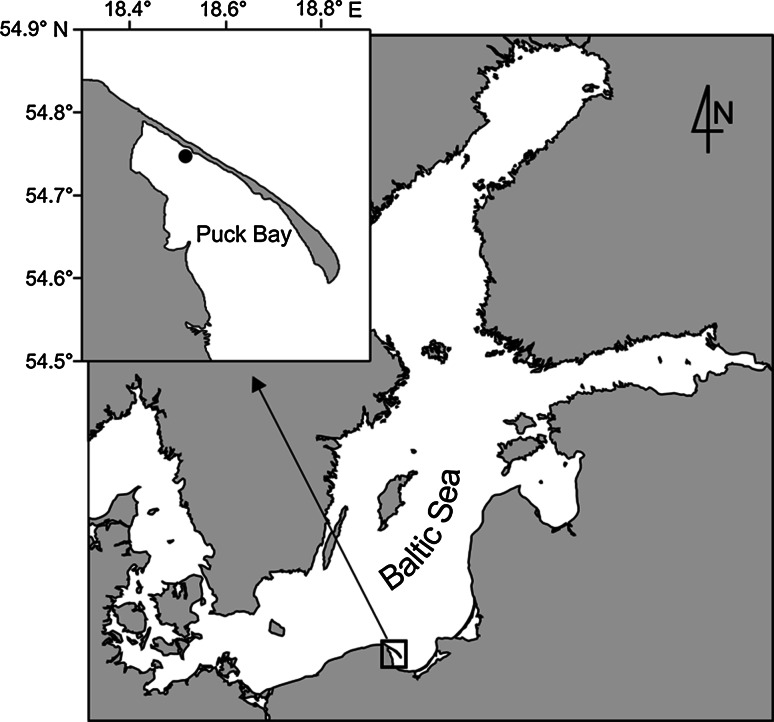
Map of the investigation area

Sediment and fauna for the experiment were collected from a sheltered site in the inner part of Puck Bay (Polish Baltic coast) (Fig. 1), near Chałupy, at 70–80 cm water depth.

Sediment granulometry was determined on each of three replicate samples, taken with cores with an internal diameter of 3.6 cm and a surface area of 10 cm^2^. Analysis was done by standard dry sieving and the sediment fractions then defined according to the Wentworth scale. Sediment parameters including median grain size, sorting coefficient and percentage contribution of the sediment fractions were calculated in the software GRADISTAT (Blott and Pye 2001).

Three sediment cores, 1.13 cm^2^ in surface area, were also taken to determine carbon, nitrogen and phytopigment (Chl*a* and phaeopigments) concentrations. These samples were stored frozen (at −20 °C) until analysis. Organic carbon (C_org_) and total nitrogen (N_tot_) concentrations were determined on dried samples by thermal combustion using a CHN analyzer (Perkin Elmer 2400). Samples for C_org_ were pre-treated with HCl to remove carbonates. Sediment phytopigments were measured fluorometrically with the Turner Designs Fluorometer after extraction with 90 % acetone for 24 h at 4 °C in the dark. Phaeopigments concentration was measured after acidification with 10 % HCl.

For porewater ammonium content, three cores with an inner diameter of 3.6 cm were collected and frozen at −20 °C. The day before the analysis the samples were thawed at room temperature. Porewater was extracted from the sediment by the vacuum method using a Nalgene filtration unit with Whatman GF/F filters. Ammonium concentrations were measured immediately after extraction according to standard methods recommended for the Baltic Monitoring Programme in online diluted samples (Grasshoff et al. 1983; HELCOM 1988).

### Sediment and fauna sampling

The experiment was performed in September 2009. Triplicate sediment cores with an internal diameter of 3.6 cm and a surface area of 10 cm^2^ were collected randomly at the study site to determine the abundance and structure of the meiobenthic community, hereafter referred to as the field community. Sediment cores were sliced immediately on sampling into seven depth layers: 0–1, 1–2, 2–3, 3–4, 4–5, 5–10, 10–15 cm. These slices were preserved separately in neutral 4 % formaldehyde and were further processed in accordance with methods described below for meiofauna in the experiment. Nematode densities and assemblage structure of the field community served as a field control. Sediment for the experiment was taken to a depth of 10 cm using a hand-held core with a 225-cm^2^ surface area. The sediment was immediately sieved over 1-mm mesh in a small amount of ambient seawater in order to exclude macrofauna, but retain the interstitial biota. This may have also excluded larger meiofauna, and although we acknowledge this is not ideal, it was felt to be the most replicable method of eliminating the macrofaunal component. In the laboratory, the sediment was gently homogenized by hand and put into plexiglass cores, with a 12.3-cm internal diameter and 33 cm long, to a depth of 15 cm. These sediment cores were then placed into a water bath consisting of two tanks with a total volume of 1,100 dm^3^ and connected to a reservoir of 2,400 dm^3^ equipped with a cooling system and supplied with an open-loop seawater pumping system. Water for the system was pumped directly from the sea and then transported to the laboratory at a distance of 50 m. Prior to entering the system, the seawater was filtered on a 2-mm mesh to remove large organic material and fauna, thus the water contained natural concentrations of phytoplankton and other organic suspension. Water was distributed among cores via plastic tubes to facilitate turnover of the overlying water (15 cm deep). Outflow water was recycled. Water in the whole system was replaced with fresh seawater once a week. The sediment was allowed to stabilize for 14 days before the macrofauna was added. A preliminary experiment performed with the same sediment and community as those used in this study showed that this period was sufficient to allow the meiofauna to re-establish its vertical distribution with the majority of organisms in the top sediment layer (on average 85 % in the upper 3 cm vs. 77 % in the field). The average total meiofauna abundance of 1,334 ind. 10 cm^−2^ recorded after the stabilization period was in the ranges noted in the field (934–2,450 ind. 10 cm^−2^).

Shallow benthic environments of the Puck Bay are strongly dominated by the polychaete *Hediste diversicolor* and bivalves. Three species differing in terms of their lifestyle and feeding behaviour were selected for the study: *Hediste diversicolor*, *Mya arenaria* and *Cerastoderma glaucum*, hereafter referred to in the text as *Hediste, Mya, Cerastoderma* and abbreviated to H, M, C, in figures and tables, respectively.

Filter- and/or surface deposit-feeding *H. diversicolor* is a burrowing species building a system of galleries extending to a depth of 20 cm. It feeds either by filtering organic suspensions from the water in its burrow or by swallowing surface sediment as well as plant and animal remains around the burrow opening (Kristensen 2001). The two bivalves, *Cerastoderma* and *Mya,* are both suspension feeders and pump water by extending their siphons to the sediment surface, but their bioturbatory behaviours considerably differ. The cockle *Cerastoderma* has two short and separate siphons, lives actively near the sediment surface (2–4 cm deep) acting as a biodiffuser mixing surface sediment particles. The soft-shell clam *Mya* has only one large and long siphon (its two siphons are fused), buries relatively deeply (10–25 cm) and leads a sessile lifestyle. Due to leakage of water from the shell, the latter species can have a profound effect on sediment biochemistry (Hansen et al. 1996) and its observed animal-sediment interaction may resemble those found for polychaete burrows (Forster and Zettler 2004).

Only intact specimens, within a specified average size range typical for the natural conditions, were chosen for the experiment: *Cerastoderma* (9–15 mm, 0.2–1 g w.wt), *Hediste* (40–70 mm long, 0.3–0.5 g w.wt.), *Mya* (15–25 mm, 0.3–1.2 g w.wt.). They were added to the microcosms in the following combinations: 0, experimental control (no macrofauna); H, *Hediste*; C, *Cerastoderma*; M, *Mya*; H + C, *Hediste* + *Cerastoderma*; H + M, *Hediste* + *Mya*; C + M, *Cerastoderma* + *Mya*. Each treatment was performed with three replicate cores.

The macrofaunal densities added to the microcosms (Table 1) ranged from 280 to 610 ind. m^−2^ (*Hediste*), 450–610 ind. m^−2^ (*Cerastoderma*) and 170–280 ind. m^−2^ (*Mya*). These densities corresponded to the average density ranges recorded at the study site (i.e. *Hediste*: 267–1,067 ind. m^−2^, *Cerastoderma*: 178–889 ind. m^−2^, *Mya*: 15–340 ind. m^−2^) (Gromisz, unpubl.), although the densities of bivalves were from the upper range of their typical natural densities, whilst the numbers of the polychaete were within the lower range of its natural abundance. Specimens were added to the microcosms to obtain in all treatments similar total macrobenthic biomass (2.1 ± 0.17 g w.wt. per core) which was close to that recorded in the field during the study period, i.e., a mean of 170 g w.wt. m^−2^. In two-species mixtures, species were added in equal weight proportions (Table 1). Wet weight of bivalves excluded water inside the valve (30 % of total bivalve weight, Pers. Obs.), but included shell weight to express the potential effect of the biological activity and physical disturbance. This approach is broadly similar to that used in studies focused on the effect of macrobenthic diversity and functional traits on ecosystem processes (Biles et al. 2003; Ieno et al. 2006; Norling et al. 2007). Shell-free weight of the bivalves appropriate for the experiment would result in unnaturally high densities.

**Table 1:** The mean number (and SD), dry weight (DW in grams) and shell-free dry weight (SFDW in grams) of each species added to the microcosms (*H* *Hediste,* *C* *Cerastoderma*, *M* *Mya*, *H* *+* *C* *Hediste* + *Cerastoderma*, *C* *+* *M Cerastoderma* + *Mya*, *H* *+* *M* *Hediste* + *Mya*)

Treatment	H				C				M				Total		
	Ind. core^−1^	Ind. m^−2^	WW core^−1^	DW core^−1^	Ind. core^−1^	Ind. m^−2^	WW core^−1^	SFDW core^−1^	Ind. core^−1^	Ind. m^−2^	WW core^−1^	SFDW core^−1^	Ind. core^−1^	WW core^−1^	DW/SFDW core^−1^
Control	–	–	–	–	–	–	–	–	–	–	–	–	–	–	–
H	7.3 (0.58)	610	2.1 (0.08)	0.29 (0.01)	–	–	–	–	–	–	–	–	7.3	2.1	0.29
C	–	–	–	–	7.3 (0.58)	610	2.1 (0.01)	0.18 (0.002)	–	–	–	–	7.3	2.1	0.18
M	–	–	–	–	–	–	–	–	3.3 (0.58)	280	2.1 (0.02)	0.18 (0.004)	3.3	2.1	0.18
H + C	3.3 (0.58)	280	1.2 (0.2)	0.14 (0.03)	5.3 (0.58)	450	1.0 (0.01)	0.09 (0.001)	–	–	–	–	8.6	2.2	0.23
C + M	–	–	–	–	5.3 (0.58)	450	0.9 (0.18)	0.08 (0.02)	2 (0.00)	170	1.2 (0.05)	0.10 (0.03)	7.3	2.1	0.18
H + M	3.7 (0.58)	310	1.0 (0.04)	0.12 (0.005)	–	–	–	–	2 (0.00)	170	1.1 (0.13)	0.10 (0.01)	5.7	2.1	0.22

DW and SFDW were calculated based on live wet weight using conversion factors (DW of *Hediste*: Wetzel et al. 2005; SFDW of bivalves: Ricciardi and Bourget 1998)

Within half an hour of adding the specimens, the majority had buried into the sediment. It was assumed that specimens which had not buried within this time were dead or damaged and these were replaced by another specimen. The microcosms were then incubated at 14 °C for 1 month. During this time, each microcosm was monitored twice per day to control water temperature and overlying water exchange rate, and to remove and replace (with an animal of similar size) any dead macrofaunal specimens appearing on the sediment surface.

### Meiofauna

One sediment core with an internal diameter of 3.6 cm and a cross-sectional area of 10 cm^2^ was sampled randomly from each microcosm for the determination of meiofaunal community composition and structure. Sediment cores were sliced immediately into seven depth layers: 0–1, 1–2, 2–3, 3–4, 4–5, 5–10, 10–15 cm and these slices were preserved separately in neutral 4 % formaldehyde. Prior to meiofauna analysis, sediments were rinsed over a 1-mm mesh to remove macrofauna. Meiofauna was extracted by re-suspending the sediment and decanting the overlying water 10 times over a 38-μm mesh sieve. The fraction retained on the sieve was stained with Rose Bengal and preserved in 4 % formaldehyde. Meiofauna in each sample was counted and identified to higher taxon level under a stereo microscope. The first 120 nematodes encountered in each sample were extracted and mounted on permanent glycerine slides following the procedure described by Vincx (1996). Nematodes were identified to species or putative species using Platt and Warwick (1983, 1988), Warwick et al. (1998) and the primary literature. Wieser’s (1953) classification was used in order to distinguish four trophic groups: selective (1A) and non-selective (1B); deposit feeders, epistrate feeders (2A) and predators/omnivores (2B).

Diversity measures were calculated for the nematode species abundance data across the integrated sediment column (0–15 cm). As a fixed number of individuals were identified, several different diversity measures were calculated in order to compare species richness and diversity between treatments. Diversity was expressed by the Margalef’s species richness (*d*), Pielou’s evenness (*J*′), Shannon-Wiener diversity index (*H*′), Hill’s index (*N*_1_) and the rarefaction index ES (*x*) (Expected number of Species). One knot of 50 was used (ES(50)) to allow comparisons between different treatments. In order to compare diversity among treatments, the number of nematodes used for calculating the diversity indices was standardized to 30 % of the total number of nematodes in the microcosm (i.e. the minimum percentage of nematodes sorted for any core). This was done by randomly selecting 30 % of individuals from all nematodes recorded in all depth layers in a given microcosm.

The effect of treatment on the densities of meiofauna and selected meiofaunal major taxa, nematode trophic composition (numerical contribution of various trophic groups) and diversity in the integrated sediment column was studied by one-way PERMANOVA with factor Treatment (TR) fixed with 7 levels. The same procedure was used to test for differences in nematode densities between the experimental treatments and field community. PERMANOVA was performed using 9,999 permutations.

The effect of treatment on the vertical profiles of total meiofauna and nematode densities, and nematode community composition and structure were investigated using a three-way crossed PERMANOVA. The experimental design consisted of 3 factors: Treatment (TR) (fixed with 7 levels), sediment Depth (DE) (fixed with 7 levels) and Replicate (RE) (random factor with 3 levels) nested within Treatment (TR). Nesting the replicates in Treatment follows from the fact that different sediment layers originated from the same microcosm violating the assumption of independency of data. Multivariate analyses of nematode community structure and composition were performed on both standardized and raw untransformed, square- and fourth-root-transformed nematode genera abundance data to discriminate between the effects of macrobenthos on more common and rare genera (stronger transformations limiting the influence of dominant species in the results of analyses). A Euclidean distance and Bray-Curtis-based resemblance matrix was used for univariate and multivariate data, respectively. Since the Bray-Curtis similarity measure is undefined for two empty samples (sediment slices with no meiofauna), we used the zero-adjusted Bray-Curtis resemblance matrix for which a dummy species is added to all samples in the original abundance matrix. PERMANOVA was conducted using 9,999 permutations of residuals under a reduced model.

Significant interaction effects were further investigated using a posteriori pair-wise comparisons of factor TR within levels of DE × TR. Pair-wise tests were based on a *P* value calculated using the 9,999 Monte-Carlo permutations procedure (i.e. P(MC)). As PERMANOVA is sensitive to differences in dispersion, PERMDISP was performed to test for homogeneity of multivariate dispersion across groups. Distances of group members to the group centroids were tested by permutation within RE (TR) groups (averaged Depths) and in TR × DE groups (averaged Replicates).

Analysis of similarity percentages (SIMPER) was performed to determine the contribution of individual species to the average Bray-Curtis dissimilarity between treatments. Non-metric multi-dimensional scaling ordination (MDS) was applied to visualize the similarities between the treatments and replicates. SIMPER and PERMANOVA analyses were carried out using the software package PERMANOVA + for PRIMER (Anderson et al. 2008).

## Results

### Characteristics of the study site

Sediment at the study site had a median grain size of 375 μm. It was a well-sorted sand dominated by the medium fraction (on average 80 %), followed by fine (9 %) and coarse fractions (9 %). Organic carbon and total nitrogen content averaged 0.06 and 0.02 %, respectively, whilst sediment chlorophyll *a* and phaeophytin concentrations were 1.8 and 0.7 μg g^-1^ dry sediment, respectively. Porewater ammonium concentrations reached a maximum of 10 μmol dm^−3^ (vs. 2 μmol dm^−3^ in the overlying water) and indicated relatively good oxygen conditions in the sediment.

### Survival of macrobenthos in microcosms

From frequent visual inspections during the incubation period and during sampling, it was found that all but two macrofauna specimens initially added to the microcosms were alive at the end of the experiment. The exceptions were two of the 54 *Cerastoderma* individuals, which were found dead at the sediment surface and replaced. In control cores, an oxidized surface zone of 10–15 mm was evident, below which the sediment was grey with grayish-black spots of up to 1 cm in diameter. In the *Cerastoderma* treatments, the depth range of the oxidized zone was not visibly different, whilst in the *Hediste* treatments, both in monoculture and in mixtures with bivalves, the sediment surrounding the worm burrows was clearly oxygenated to a depth of 10–12 cm often forming a relatively uniform sediment layer. Microcosms with *Mya* in monoculture and in mixture with *Cerastoderma* either had no clearly visible changes to the oxidized zone or it extended much deeper, up to 10 cm depth. Slicing the sediment from the microcosms into layers at the end of the experiment demonstrated that *Hediste* penetrated the whole sediment column (0–15 cm), whilst *Mya* stayed in the upper 10 cm and *Cerastoderma* in the upper 2 cm.

### Meiobenthic community

**Fig. 2:**
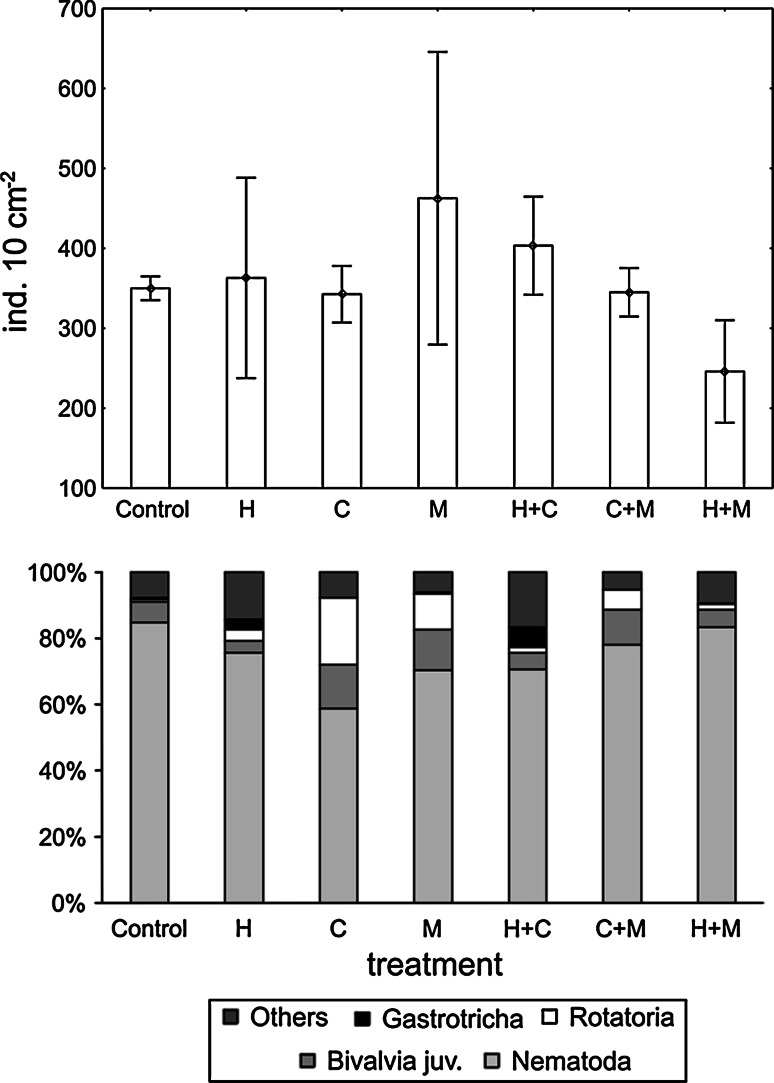
Average total meiofaunal abundance (mean ± SE) and percentage contribution of the dominant major taxa in each treatment (*H Hediste,* *C* *Cerastoderma*, *M* *Mya*, *H* *+* *C* *Hediste* + *Cerastoderma*, *C* *+* *M* *Cerastoderma* + *Mya*, *H* *+* *M* *Hediste* + *Mya*)

Average total abundances of meiofauna (integrated over the sediment column) in treatments with macrofauna were generally higher than in the control (442–656 ind. 10 cm^−2^ vs. 412 ind. 10 cm^−2^, respectively), with the exception of the *Hediste*/*Mya* complex, where the lowest total meiofaunal concentration was recorded (295 ind. 10 cm^−2^) (Fig. 2). These differences were, however, not statistically significant (PERMANOVA, *P*(perm) > 0.05) (Table 2). In all microcosms, the community was dominated by nematodes, ranging from 60 % of the total meiofaunal abundance in the *Cerastoderma* monoculture treatment to 85 % in the control. The numerical contribution of other major taxa was relatively low (1–6 %) except for juvenile bivalves and Rotatoria which constituted 11–20 % of total meiobenthic density in treatments with bivalves (Fig. 2). Abundances of these two meiofaunal groups varied significantly among treatments (Table 2) and their numbers in microcosms with bivalves (C, M) were significantly higher than in the control and treatments with *Hediste* (H, HC, HM) (pair-wise tests, *P* < 0.05). In contrast, nematode abundances in the integrated sediment column (167–802 ind. 10 cm^−2^, mean 360 ind. 10 cm^−2^) were not statistically different both across treatments (Table 2) and compared to the field community (270–601 ind. 10 cm^−2^, mean 447 ind. 10 cm^−2^) (PERMANOVA: MS = 14063, Pseudo-F = 0.54, *P*(perm) = 0.81).

**Table 2:** Results of PERMANOVA analysis for differences in densities of meiofauna and selected major meiofaunal taxa in the integrated sediment column among treatments

	Treatment			
	*df*	MS	Pseudo-F	*P* (perm)
Total meiofauna	6	44,427	1.08	n.s.
Nematodes	6	13,013	0.51	n.s.
juv. Bivalves	6	2,268	6.89	0.0031
Rotatoria	6	5,481	3.03	0.0137

*n.s.* not significant

PERMANOVA showed that Treatment had a significant effect on vertical occurrence of meiofauna and nematodes both when raw (results not shown) and standardized data were analysed (TR × DE effect, *P*(perm) < 0.05, Table 3).

**Table 3:** Results of PERMANOVA analysis for differences in vertical profiles of total meiobenthic, nematode and selected nematode species densities, and multivariate nematode community structure among treatments

	Treatment				Depth				Treatment * depth			
	*df*	MS	Pseudo-F	*P*	*df*	MS	Pseudo-F	*P*	*df*	MS	Pseudo-F	*P*
Total meiofauna	6	6,347	1.47	n.s.	6	3,758	134	0.0001	36	13,279	4.75	0.0001
Nematodes	6	1,859	0.77	n.s.	6	18,131	116	0.0001	36	6,622	4.25	0.0002
Untransformed	6	12,013	2.77	0.0076	6	15,697	8.48	0.0001	36	3,041	1.63	0.0002
√ rt-transformed	6	11,682	3.30	0.0023	6	14,894	10.57	0.0001	36	2,338	1.66	0.0001
4th rt-transformed	6	11,205	4.15	0.0018	6	14,841	14.36	0.0001	36	1,744	1.69	0.0001
*A. thalassophygas*	6	311	0.31	n.s.	6	1,790	4.69	0.0006	36	365	0.96	n.s.
*A. elongatus*	6	1,517	1.56	n.s	6	1,076	2.72	0.0195	36	909	2.29	0.0012
*A. viviparum*	6	177	6.45	0.0037	6	172	15.91	0.0001	36	2,536	2.34	0.0013
*D. zeelandicus*	6	293	2.46	n.s.	6	952	11.35	0.0001	36	196	2.34	0.0012

*n.s.* not significant effect

*P* values obtained by permutation

PERMDISP tests showed, however, that there was no homogeneity of multivariate dispersion (*P*(perm) < 0.05) in TR × DE groups (both standardized and raw data, and all data transformations). Examination of the patterns in the ordination plots revealed that levels of factor DE representing top sediment layers were less dispersed than other levels regardless of the treatment. PERMDISP showed that there was homogeneity of multivariate dispersion (*P*(perm) > 0.05) in TR × DE groups when the three levels of factor DE (0–1, 1–2, 2–3 cm) were excluded from the analysis (both standardized and raw data). Pair-wise tests revealed significant differences in nematode densities in 3–4, 4–5, 5–10 and 10–15 cm layers between *Hediste* microcosms and other treatments, whereas no differences were found among bivalve treatments (Table 4).

**Table 4:** Results of pair-wise tests for differences in nematode densities (standardized, √rt-transformed) across treatments (TR) within levels of factor depth (DE) (*H* *Hediste,* *C* *Cerastoderma*, *M* *Mya*, *H* *+* *C* *Hediste* + *Cerastoderma*, *C* *+* *M* *Cerastoderma* + *Mya*, *H* *+* *M* *Hediste* + *Mya*)

Sediment depths	Treatments, P(MC) < 0.05
3–4	H, Control
	H + C, Control
	H, C + M
	H + C, C
	H + C, C + M
	H + M, C + M
4–5	H, C
	H + C, C
	H + M, C
5–10	Control, C + M
	H, C
	H, M
	H, C + M
	H + C, C
	H + C, M
	H + M, M
	H + C, C + M
	H + M, C + M
10-15	H, Control
	H, C
	H, M

*P(MC)* Monte-Carlo permutations. Non-significant effects not shown

The dispersions in RE (TR) groups were always homogenous (PERMDISP, *P*(perm) > 0.05). Pair-wise tests showed significant differences in nematode community structure (all data transformations) between all *Hediste* microcosms and other treatments (an exception H + M and M), whereas no differences were found among bivalve treatments and between bivalve treatments and the defaunated control (Table 6).

**Fig. 3:**
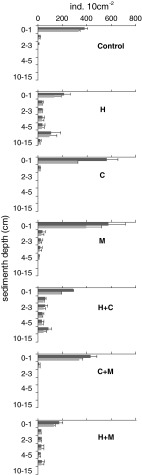
Vertical profile of total meiofaunal and nematode abundance (mean ± SE) across the different treatments (*H Hediste,* *C* *Cerastoderma*, *M* *Mya*, *H* *+* *C* *Hediste* + *Cerastoderma*, *C* *+* *M* *Cerastoderma* + *Mya*, *H* *+* *M* *Hediste* + *Mya*). *Black columns* meiobenthos, *grey columns* nematodes

In all treatments where *Hediste* was present, both in monoculture and in combinations with other species, meiofauna penetrated deeper sediment layers (up to 10–15 cm) and only 49 % (*Hediste* treatment)—63 % (*Hediste*/*Mya* treatment) of the total meiofaunal community was recorded at the sediment surface. In contrast, in all other treatments, including the control, the majority of meiofauna (90 % in the *Mya* treatment to 97 % in the *Cerastoderma*/*Mya* treatment) was concentrated in the upper one centimetre of the sediment (Fig. 3). In *Hediste* treatments, the deeper sediment layers were strongly dominated by nematodes, constituting up to 100 % of the total meiofaunal abundance. Vertical occurrence of nematodes in the field also extended to deeper sediment layers. On average, 77 % of nematodes were recorded in the top 3 cm, whilst almost one quarter of the assemblage penetrated the sediment to a depth of 10-15 cm (Fig. 3).

In total 25 nematode genera were recorded (Table 5), with 21 putative species identified and a further 4 multi-species genera. The species in these latter genera were relatively rare and it was not possible to differentiate them owing to a paucity of male specimens. All the species recorded in the field were also recorded in the microcosms at the end of the experiment (Table 5). PERMANOVA performed on nematode genera presence/absence data from the experiment and from the field showed, however, significant differences in the nematode composition among treatments (MS = 327, Pseudo-F = 2.84, *P*(perm) = 0.0013). Pair-wise tests found that the composition of the field community differed significantly from those in the *Cerastoderma*-only, *Mya*-only and experimental control (*P*(perm) < 0.05), but did not differ from the assemblages in *Hediste*-only and all two-species mixtures (Table 6).

**Table 5:** Average percentage abundance of nematode taxa in the field and in each treatment (mean ± SE) (*H* *Hediste,* *C* *Cerastoderma*, *M* *Mya*, *H* *+* *C* *Hediste* + *Cerastoderma*, *C* *+* *M* *Cerastoderma* + *Mya*, *H* *+* *M* *Hediste* + *Mya*)

Species	Tropic group	Field data	Treatments						
			Control	H	C	M	H + C	C + M	H + M
*Adoncholaimus thalassophygas*	2B	19.9 ± 3.5	42.6 ± 7.1	24.6 ± 3.4	28.9 ± 2.8	41.6 ± 5.6	19.5 ± 1.7	30.3 ± 7.2	19.9 ± 2.5
*Anoplostoma viviparum*	1B	8.6 ± 3.3	4.7 ± 1.4	10.6 ± 0.5	5.4 ± 2.9	3.7 ± 1.2	9.2 ± 0.6	5.1 ± 1.0	8.3 ± 2.2
*Ascolaimus elongatus*	1B	1.2 ± 0.8	2.2 ± 1.4	11.7 ± 0.6	1.9 ± 0.6	2.2 ± 0.8	9.8 ± 2.7	0.4 ± 0.3	7.2 ± 2.4
*Axonolaimus spinosus*	1B	4.1 ± 0.6	4.0 ± 2.6	3.8 ± 1.9	7.3 ± 2.0	6.6 ± 1.1	5.9 ± 0.7	8.2 ± 1.9	7.0 ± 1.9
*Calomicrolaimus* cf. *honestus*	2A	5.2 ± 2.7	0.2 ± 0.2	3.6 ± 0.8			4.7 ± 1.2	1.9 ± 1.1	3.6 ± 0.4
*Chromadorita* spp.	2A	1.7 ± 0.5	1.7 ± 0.5	1.4 ± 0.8	3.1 ± 1.4	1.7 ± 0.0	2.8 ± 0.2	1.8 ± 0.1	3.0 ± 1.6
*Daptonema* aff. *setosus* sp. A	1B	2.2 ± 0.4		0.2 ± 0.1	1.1 ± 0.5	0.5 ± 0.5	1.0 ± 0.4	0.5 ± 0.2	0.8 ± 0.3
*Desmolaimus* cf. *zeelandicus*	1B	11.9 ± 2.7	15.4 ± 2.7	6.8 ± 0.4	15.2 ± 4.5	8.1 ± 2.1	9.6 ± 1.4	13.8 ± 7.4	17.0 ± 4.9
*Dichromadora cephalata*	2A	0.5 ± 0.2	0.3 ± 0.2	0.9 ± 0.3			0.7 ± 0.3		0.1 ± 0.1
*Diplolaimella* sp. 1	1B	0.1 ± 0.1	0.1 ± 0.1						
*Dorylaimus* sp. 1	*2B*		0.1± 0.0						
*Eleutherolaimus* cf. *stenosoma*	1B	0.3 ± 0.2							0.2 ± 0.2
*Enoplolaimus* spp.	2B	0.5 ± 0.2	0.5 ± 0.2	2.6 ± 0.7	1.1 ± 1.1	3.3 ± 2.1	3.5 ± 0.8	3.1 ± 2.3	4.0 ± 1.0
*Enoplus* aff. *brevis*	2B	0.5 ± 0.2	0.9 ± 0.8	0.2 ± 0.1	0.7 ± 0.8	0.1 ± 0.0	0.7 ± 0.1	0.1 ± 0.0	1.5 ± 0.8
*Halomonhystera disjuncta*	1B	0.2 ± 0.1	3.0 ± 0.6	0.8 ± 0.5	9.2 ± 1.7	12.2 ± 1.7	0.4 ± 0	8.0 ± 2.7	0.9 ± 0.3
*Hypodontolaimus* spp.	2A	9.7 ± 1.1	2.4 ± 0.4	2.3 ± 0.8	1.9 ± 0.1	1.0 ± 0.5	3.6 ± 0.4	0.8 ± 0.8	2.6 ± 0.3
*Leptolaimus papilliger*	1A	0.2 ± 0.2		0.7 ± 0.3	0.3 ± 0.2	0.2 ± 0.5	0.6 ± 0.1	0.4 ± 0.2	0.5 ± 0.0
*Oncholaimus oxyuris*	2B	9.0 ± 1.1	5.0 ± 1.7	12.2 ± 3.9	6.3 ± 1.1	5.6 ± 1.5	7.1 ± 0.9	10.0 ± 0.5	5.4 ± 2.1
*Paracanthonchus* spp.	2A	2.1 ± 0.6	0.9 ± 0.8	5.7 ± 2.2	3.2 ± 1.5	1.5 ± 0.4	4.8 ± 0.6	2.0 ± 0.5	3.9 ± 0.8
*Paracyatholaimus proximus*	2A	6.8 ± 2.9	7.6 ± 3.6	3.0 ± 0.4	7.6 ± 2.2	4.6 ± 2.6	6.7 ± 1.3	5.0 ± 2.6	4.5 ± 3.4
*Prochromadorella* sp. 1	2A	0.1 ± 0.1			0.3 ± 0.2				
*Sphaerolaimus* cf. *balticus*	2B	3.9 ± 0.5	6.0 ± 2.1	6.7 ± 1.7	5.3 ± 1.4	2.2 ± 1.1	7.5 ± 0.6	6.5 ± 3.3	6.9 ± 1.3
*Theristus flevensis*	1B	2.9 ± 1.2	1.9 ± 1.2	1.2 ± 0.4	1.4 ± 0.2	3.2 ± 3.8	1.2 ± 0.1	0.8 ± 0.4	1.2 ± 0.1
*Tripyloides marinus*	1B	6.6 ± 1.3		0.2 ± 0.1		1.6 ± 0.5	0.2 ± 0.1	0.3 ± 0.2	0.1 ± 0.1
*Viscosia viscosia*	2B	1.8 ± 0.6		0.2 ± 0.3			0.1 ± 0.0	0.3 ± 0.2	
Indet.			0.5 ± 0.1	0.6 ± 0.2	0.1 ± 0.0	0.2 ± 0.0	0.4 ± 0.1	0.7 ± 0.2	1.3 ± 0.2

Wieser’s trophic groups are indicated for each taxa. Mean ± SE

**Table 6:** Results of pair-wise tests for differences in nematode community structure across treatments (*F* field community, *H* *Hediste,* *C* *Cerastoderma, M* *Mya,* *H* *+* *C* *Hediste* + *Cerastoderma,* *C* *+* *M* *Cerastoderma* + *Mya,* *H* *+* *M* *Hediste* + *Mya*)

Pairs of treatments	Presence/absence		Untransformed		√rt		4th rt	
	*t*	*P*	*t*	*P*	*t*	*P*	*t*	*P*
F, Control	2.52	0.0316	2.08	0.0401	2.13	0.0341	2.28	0.0251
F, H		n.s.	2.10	0.0286	2.17	0.0233	2.00	0.0381
F, C	3.47	0.0046	1.95	0.0403	2.39	0.0166	2.80	0.009
F, M	2.90	0.019	2.38	0.0205	2.47	0.0172	2.60	0.0148
F, H + C		n.s.	1.99	0.0358	2.33	0.0202	2.38	0.0193
F, C + M		n.s.		n.s.		n.s.		n.s.
F, H + M		n.s.		n.s.	2.08	0.0316	2.19	0.0228
H, Control	3.09	0.0035	2.05	0.0081	2.27	0.0053	2.61	0.003
H + C, Control	3.29	0.0029	1.86	0.0221	2.08	0.0144	2.52	0.0058
H + M, Control	2.28	0.0165	1.63	0.0435	1.81	0.028	2.00	0.0186
H, C	4.13	0.0017	2.40	0.0051	2.77	0.0025	3.23	0.0014
H, M	2.22	0.042	1.94	0.0305	2.04	0.026	2.14	0.0315
H, C + M	4.75	0.0003	3.76	0.0001	3.78	0.0002	4.23	0.0002
H + C, C	4.85	0.0006	2.17	0.0131	2.61	0.0046	3.38	0.0013
H + C, M	2.23	0.0406		n.s.		n.s.	2.1	0.0429
H + C, C + M	5.71	0.0003	3.12	0.0005	3.65	0.0003	4.42	0.0002
H + M, C	2.86	0.0074	2.03	0.0107	2.24	0.0075	2. 51	0.0054
H + M, C + M	3.37	0.0025	2.77	0.0006	3.00	0.0008	3.17	0.0006
H + M, M		n.s.		n.s.		n.s.		n.s.
H, H + C		n.s.		n.s.		n.s.		n.s.
H, H + M		n.s.		n.s.		n.s.		n.s.
H + C, H + M		n.s.		n.s.		n.s.		n.s.
C, Control		n.s.		n.s.		n.s.		n.s.
M, Control		n.s.		n.s.		n.s.		n.s.
C + M, Control		n.s.		n.s.		n.s.		n.s.
C, M		n.s.		n.s.		n.s.		n.s.
C, C + M		n.s.		n.s.		n.s.		n.s.
M, C + M		n.s.		n.s.		n.s.		n.s.

*n.s.* not significant

*P* values obtained by permutation;

Predators/omnivores (2B) followed by non-selective deposit feeders (1B) dominated all treatments, including the control. Predators/omnivores were generally dominated by *Adoncholaimus thalassophygas* and *Oncholaimus oxyuris* followed by *Sphaerolaimus* cf*. balticus*, together accounting for 49–60 % (with an average of 55 %) of the total nematode community in the control and 38–53 % in treatments with macrofauna. The contribution of non-selective deposit feeders ranged between 32 % in the control and 36–43 % in macrofauna treatments: *Desmolaimus* cf. *zeelandicus* was dominant across all treatments, additionally *Anoplostoma viviparum* and *Ascolaimus* cf. *elongatus* were co-dominant in the *Hediste* treatments, and *Halomonhystera disjuncta* was co-dominant in the non-*Hediste* treatments. Epistrate feeders (2A) constituted between 9 % (*Mya*-only treatment) and 24 % (*Hediste/Cerastoderma* treatment) of the total nematode abundance. Selective deposit feeders (1A) were represented only by one species, *Leptolaimus papilliger*, which was recorded in all treatments with macrofauna, but at <1 % of total abundance, and was not recorded in the control. The differences across treatments in nematode trophic composition were not statistically significant (PERMANOVA, *P*(perm) > 0.05).

**Fig. 4:**
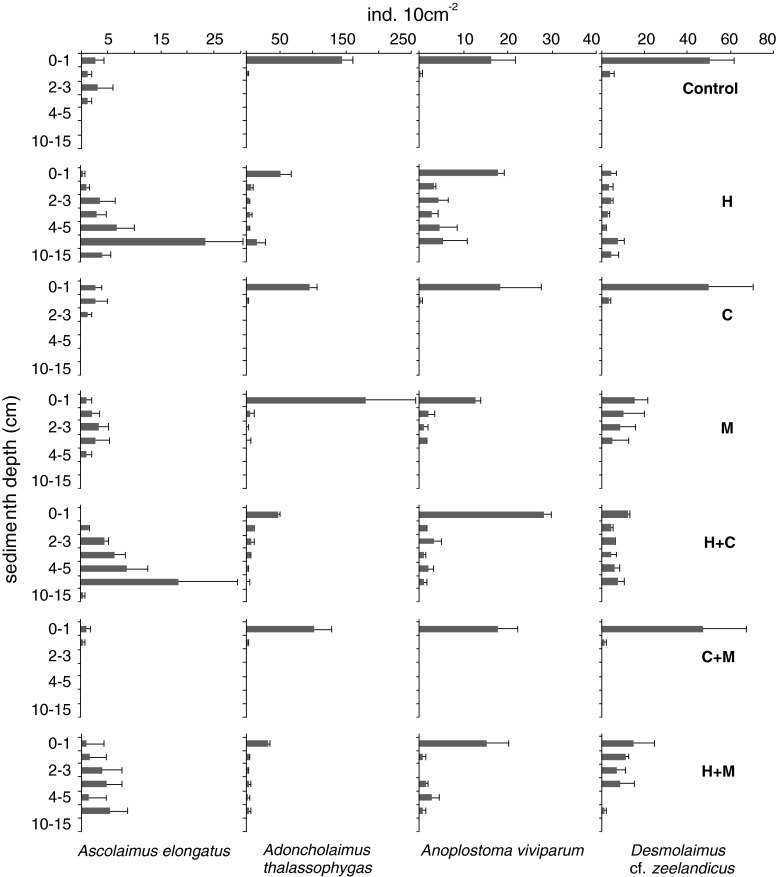
Vertical distribution of selected dominant nematode species across the different treatments (*H Hediste,* *C* *Cerastoderma*, *M* *Mya*, *H* *+* *C* *Hediste* + *Cerastoderma*, *C* *+* *M* *Cerastoderma* + *Mya*, *H* *+* *M* *Hediste* + *Mya*) (mean ± SE)

The percentage dominance of the abundant *A. thalassophygas* was reduced in treatments with *Hediste* compared to other treatments (20–25 vs. 29–43 %), but PERMANOVA showed no effect of treatment on either total densities or vertical distribution of this species (Table 3). In contrast, *A. viviparum* and *A. elongatus* were more abundant in *Hediste* microcosms (8.3–10.6 vs. 3.7–5.1 % and 7.2–11.7 vs. 0.4–2.2 %, respectively) and their vertical profiles were significantly affected by the treatment (Table 3). Also, the vertical distributions of the dominant nematode species extended into deeper sediment layers in all *Hediste* treatments (H, HC, HM) and in the *Mya*-only treatment (Fig. 4).

The field community was dominated by *A. thalassophygas* and *D.* cf. *zeelandicus* together accounting for 32 % of the whole nematode assemblage. The deepest sediment layers were dominated by *C*. cf. *honestus*, and *Desmolaimus* cf. *zeelandicus.*

**Fig. 5:**
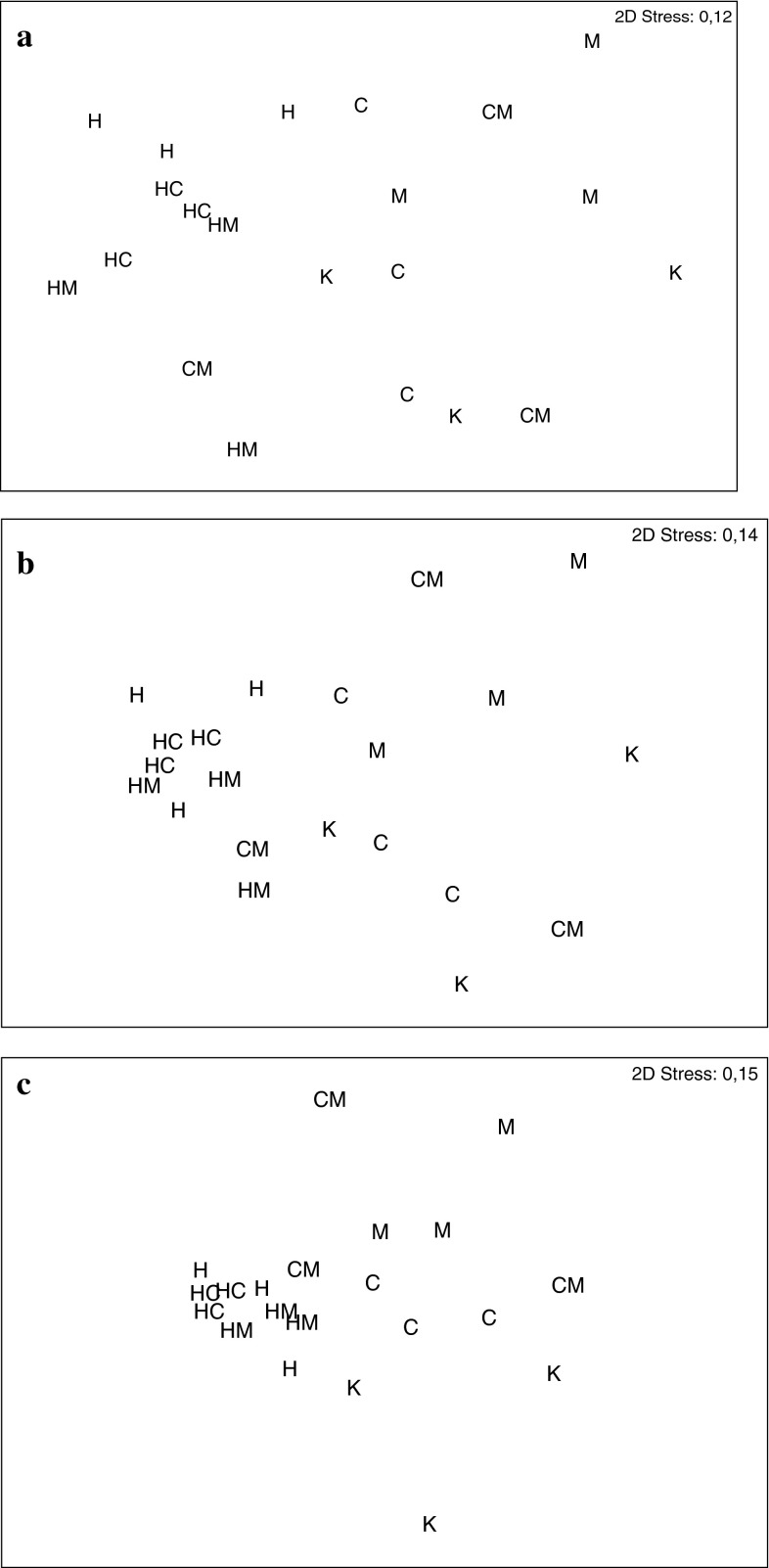
Non-metric multi-dimensional scaling (MDS) ordinations of treatment similarity based on standardized **a** untransformed, **b** square-root and **c** fourth-root transformed nematode genera abundance data (*K* Control, *H* *Hediste,* *C* *Cerastoderma*, *M* *Mya*, *H+C Hediste* + *Cerastoderma*, *C+M* *Cerastoderma* + *Mya*, *H+M* *Hediste* + *Mya*)

Similarities of nematode assemblages between and within *Hediste* treatments, both in monoculture and in mixture, were higher than similarities within any other single treatment and this effect was more pronounced with increasing data transformation (Table 7, Fig. 5).

**Table 7:** Similarities between/within selected treatments obtained from PERMANOVA analysis for standardized √- and 4th rt-transformed nematode genera abundance data (*H* *Hediste,* *C* *Cerastoderma*, *M* *Mya*, *H* *+* *C* *Hediste* + *Cerastoderma*, *C* *+* *M* *Cerastoderma* + *Mya*, *H* *+* *M* *Hediste* + *Mya*)

Treatment	√	4th rt
Control & control	26	35
H & H	49	54
C & C	33	41
M & M	33	39
H & H + C	49	54
H & H + M	42	47
H + C & H + C	47	52
H + M & H + M	37	43
H + C & H + M	41	46
C + M & C + M	35	43

According to SIMPER analysis, the abundant *Ascolaimus elongatus*, *Halomonhystera disjuncta* and *Adoncholaimus thalassophygas* and the relatively rare *Calomicrolaimus* cf. *honestus* were largely responsible for dissimilarity between nematode assemblages recorded with the polychaete and other treatments (Table 8).

**Table 8:** Selected between-treatment dissimilarity percentages of nematode communities

Treatment	Genera responsible for dissimilarity
Control & H	*Ascolaimus, Paracanthonchus*
Control & H + C	*Adoncholaimus, Calomicrolaimus*
Control & H + M	*Adoncholaimus, Calomicrolaimus*
H & C	*Halomonhystera, Ascolaimus*
H & M	*Halomonhystera, Ascolaimus*
H & C + M	*Ascolaimus, Halomonhystera*
C & H + C	*Halomonhystera, Calomicrolaimus*
C & H + M	*Halomonhystera, Calomicrolaimus*
M & H + C	*Halomonhystera, Calomicrolaimus*
H + C & C + M	*Ascolaimus, Halomonhystera*
C + M & H + M	*Ascolaimus, Halomonhystera*

Results of SIMPER analyses of square-root transformed nematode genera abundance data. The genera accounting for the majority of dissimilarity between treatments are shown (Control; *H Hediste,* *C* *Cerastoderma*, *M* *Mya*, *H* *+* *C* *Hediste* + *Cerastoderma*, *C* *+* *M* *Cerastoderma* + *Mya*, *H* *+* *M* *Hediste* + *Mya*)

**Fig. 6:**
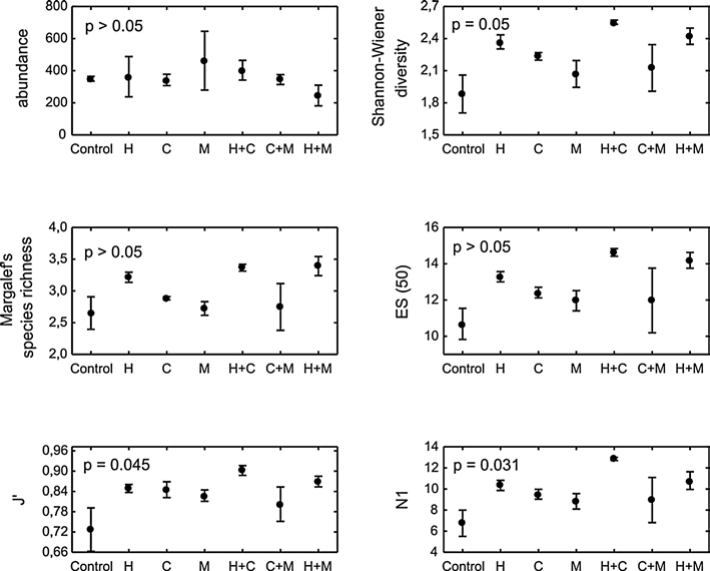
Univariate indices (mean ± SE) of nematode community structure in the different treatments (*H Hediste,* *C* *Cerastoderma*, *M* *Mya*, *H* *+* *C* *Hediste* + *Cerastoderma*, *C* *+* *M* *Cerastoderma* + *Mya*, *H* *+* *M* *Hediste* + *Mya*). *P*(perm) values derived from PERMANOVA analysis

The univariate diversity measures were always highest in *Hediste* treatments (Fig. 6), though except for Pielous evenness index and the *N*_1_ diversity index, the differences in nematode diversity among treatments were not significant (PERMANOVA, *P*(perm) > 0.05). Pair-wise tests found that evenness in the *Hediste*/*Cerastoderma* mixture differed significantly (*P*(perm) < 0.05) from those in the *Mya-*only and *Hediste-*only treatment, whilst *N*_1_ in the *Hediste/Cerastoderma* mixture differed significantly from those in the control and all single-species treatments (data not shown).

## Discussion

### Methods

Prior to the addition of macrofauna, the sediment was sieved and mixed, thus the natural vertical distribution of meiobenthos was initially destroyed. This procedure is in contrast to that used by Braeckman et al. (2011a) in a similar experiment from the North Sea. These authors sliced the sediment, sieved the slices separately and reconstructed the sediment column and natural vertical distribution of meiofauna. However, in our experiment, the vertical meiofauna distribution was re-established during the stabilization period. Results of our preliminary experiment showed that at the end of the 14-day long stabilization period the vertical distribution of nematodes was not uniform: the upper sediment layers (0–3 cm) supported the majority of nematodes (on average, 85 vs. 77 % in the field). Hence, prior to macrofauna addition we achieved a new equilibrium in vertical meiofauna distribution in our microcosms.

Nematode densities recorded in our microcosms, including in the defaunated control, were at the end of the experiment not significantly different from those recorded in the field on the day of sediment sampling, suggesting no mortality of nematodes under the experimental conditions. Analysis of the nematode species presence/absence data revealed that nematode species composition in only three out of the total of seven treatments (the control, *Cerastoderma*-only and *Mya*-only treatments) was significantly impoverished compared to the field, whilst all other treatments supported communities, in terms of their composition, similar to the field. This observation indicated that our experimental procedure, i.e., sediment sieving and homogenization, had no actual effect on nematode community composition, which was affected during the incubation period by the presence/absence and species identity and composition of macrofauna.

### Effect of macrofauna on meiofauna

There is as yet no consensus on the role of the macrobenthic species used in our experiment in structuring meiobenthic communities (for overview see Olafsson 2003): *Hediste diversicolor* has been recorded to have both a neutral (Kennedy 1993) and a negative (Reise 1979) effect on meiofaunal density. *Cerastoderma* spp. has been reported to have no influence on meiobenthic density (Reise 1983; Kennedy 1993), whilst there have been no studies on the interaction between *Mya* and meiofaunal assemblages. In our study, the addition of macrofauna did not have an impact on total meiofaunal or nematode abundances. The densities of meiofauna in treatments with macrofauna were not significantly different from those in the defaunated control and in the field indicating a lack of mortality due to either an absence of the bioturbator (experimental control) or due to predation and/or sediment disturbance caused by the bioturbator. Comparatively high nematode numbers, 802 and 613 ind. 10 cm^−2^, were recorded in two cores with macrofauna (*Mya*-only and *Hediste*-only cores, respectively) but the average densities of nematodes in macrofauna treatments were surprisingly not significantly different from those in the control. The lack of variation in nematode abundance among treatments in our experiment is in contrast to what has been observed by Braeckman et al. (2011a) in a similar experiment from the North Sea. These authors found significantly lower nematode numbers in the defaunated control indicating nematode mortality in the absence of the bioturbator, whilst the highest nematode densities in the presence of the polychaete were related to a stimulative effect of the worm on nematode survival.

The disagreement in the results of these two experiments may reflect three principle factors: 1. sediment type; 2. size and biomass of the macrobenthic species and 3. the receiving nematode community. Braeckman et al. (2011a) used fine sediment in their experiment (median grain size of 215 μm), whilst our sediment was composed of well-sorted sand of a median grain size of 375 μm. The effect of the bioturbator on sediment characteristics may presumably be more conspicuous in fine than in medium sand (e.g. Volkenborn et al. 2007), thus the effect of the bioturbator presence/absence on meiofauna may also be more distinct in finer sediments.

There is no information about the size or biomass of the macrobenthic species used by Braeckman et al. (2011a) in their experiment. It can be assumed that the macrobenthic specimens added to the microcosms were larger than our macrofauna, and due to larger body size their effect on sediment properties and associated meiobenthic community would be stronger than in our experiment. Baltic species are much smaller than those from the North Sea due to the lower salinity. Also, the receiving nematode communities strongly differ between these two experiments. Braeckman et al. (2011a) identified 80 nematode species, whilst we have recorded 21 putative species and a further 4 multi-species genera. The species in high-diversity communities are highly specialized, the interspecific connections are more complex and species niche overlap is presumably more frequent than in low-diversity system. It can be hypothesized, therefore, that high-diversity communities are more sensitive, respond more rapidly and their response to the presence/absence of the bioturbator is more pronounced than in low-diversity communities.

In our study, the addition of macrofauna did result in changes in the composition of some meiofaunal major taxa. Numbers of rotifers and juvenile bivalves were significantly enhanced in *Cerastoderma* and *Mya* treatments (Fig. 2): in sediments inhabited by adult bivalves, enhanced numbers of juvenile bivalves may reflect local facilitation of larval settlement owing to overlying water dynamics and circulation associated with bivalve siphonal currents (Ertman and Jumars 1988). Filter-feeding rotifers might have also benefited from water currents generated by bivalve filtering activity. In addition, a biofilm observed on the sediment surface composed of bivalve pseudofaeces and faeces probably encouraged microbial activity which could then sustain rotifers at the sediment surface. According to observations from freshwater benthic habitats, rotifer occurrence is positively correlated with the thickness of the sediment surface biofilm (Majdi et al. 2012). In our experiment, the numbers of rotifers and juvenile bivalves were comparatively lower in microcosms where bivalves were incubated with *Hediste*, probably due to biofilm and sediment surface disturbance associated with the food-searching activity of the polychaete.

This study demonstrated that meiofauna exhibit habitat extension due to the presence of the *Hediste* and concur with other polychaete studies performed under both laboratory and field conditions (Reise 1983; Kennedy 1993; Tita et al. 2000; Pinto et al. 2006; Braeckman et al. 2011a). In microcosms inhabited by bivalves, the distribution of meiofauna showed no difference from the defaunated control, with the majority (on average >90 % of total abundance) concentrated in the top sediment layer (0–1 cm). The only exception was one replicate core from the *Mya*-only treatment where meiofauna reached greater sediment depth: nematodes penetrated the sediment to a depth of 5 cm and reached their highest abundance (800 ind. 10 cm^−2^) compared to other replicates (174 and 412 ind. 10 cm^−2^). This variability appeared to reflect spatial patchiness in meiofauna distribution as an effect of the presence of the bivalve and associated changes in the interstitial environment. In the case of the sessile *Mya*, repeated withdrawals and extensions of the siphon and transport of water and oxygen from the shells are known to stimulate oxygenation, biotic enrichment, microbial activity and benthic processes in the thin sediment zone surrounding the bivalve (Henriksen et al. 1983; Reise 1983; Hansen et al. 1996; Forster and Zettler 2004). This specific micro-environment probably attracted nematodes (burrow effect) and was responsible for the high within-replicate variability in the *Mya* treatment. Additionally, the variation in nematode response in *Mya* treatment might reflect variability in physiological status and activity of this deeply burrowing bivalve in response to laboratory conditions.

There was, however, no overall effect of *Mya* on vertical occurrence of meiobenthos. Meiofauna vertical distribution in the *Mya* treatment was similar to those in *Cerastoderma* treatment and the control. The same was observed in treatments where both bivalves were incubated together. Addition of *Hediste* to microcosms with bivalves, however, extended the vertical distribution of meiofauna. In all treatments where *Hediste* was present, both in isolation and in mixture with bivalves (H, HC, HM), meiofauna, particularly nematodes, occupied deeper sediment depths and only 30–70 % of the total meiofaunal abundance was concentrated at the sediment surface (Fig. 3). This observation demonstrated the species-specific effect of *Hediste* on the vertical distribution of meiofauna. *H. diversicolor* is an active bioirrigator and an efficient particle reworker (Kristensen and Hansen 1999; Hedman et al. 2011): these engineering powers may facilitate meiofauna to inhabit deeper sediment layers, meiofauna potentially acting opportunistically to colonize such habitats as they are formed. The food-searching activity of *Hediste* and the associated physical disturbance of the sediment surface might also force meiofauna to leave the sediment surface and migrate downward. It is worth noting that the extension of nematodes into the deeper sediment layers observed in all *Hediste* treatments was similar to that recorded in the field, where *Hediste* co-exists with all macrobenthic species used in our experiment and other macrofauna.

In addition to the nematodes, oxygen-dependant turbellarians, harpacticoids and gastrotrichs penetrated deeper sediment layers (up to 10 cm depth) where *Hediste* was present, and reached twice the abundances recorded in other treatments (data not shown).

### Effect of macrofauna on nematode community structure

Previous studies demonstrated that different macrobenthic species play different roles in structuring nematode communities. Nematode assemblage structure reflects macrobenthic disturbance types (defined by feeding behaviour and motility) (Austen et al. 1998) and community response is dependant on macrobenthic functional traits (type of bioturbation and bioirrigation) and potentially macrofaunal ecosystem engineering (Braeckman et al. 2011a, b). The two bivalves used in our experiment are both suspension feeders, but their bioturbatory behaviours considerably differ. The cockle *Cerastoderma* lives actively near the sediment surface and acts as a biodiffuser mixing surface sediment particles, whilst the soft-shell clam *Mya* buries deeply and leads a sessile lifestyle.

Our experiment showed that these two bivalves had no influence on nematode community structure. The lack of response by the nematode community in the *Cerastoderma* treatment can be related to the bivalves restriction to the sediment surface and its known poor effect on sediment processes and porewater characteristics (Mermillod-Blondin et al. 2005).

Van Colen et al. (2012) observed a strong effect of *Cerastoderma* on benthic processes but both the sediment type (mud vs. medium sand) and presumably also the size of the *Cerastoderma* specimens used in their field experiment differed from those in our study. For instance, the size of Cerastoderma used in our experiment varied between 9-15 mm, whilst the length of Cerastoderma from the North Sea may reach several cm. In contrast, *Mya* is known to stimulate oxygenation, biotic enrichment and microbial activity in its surrounding sediment (Henriksen et al. 1983; Reise 1983; Hansen et al. 1996; Forster and Zettler 2004) and may have a significant effect on sediment processes (Hansen et al. 1996). Nevertheless, we did not observe a response by the nematode community to the presence of *Mya:* Community structure in the *Mya* treatment did not differ significantly from those recorded in the control or *Cerastoderma* microcosms. High nematode abundance and penetration into deeper sediment layers in one replicate *Mya* microcosm may, however, indicate the effect of biogenic structure on nematodes. In the case of this replicate core, *Adoncholaimus* *thalassophygas* and *Desmolaimus zeelandicus* penetrated the sediment to a depth of 5 cm, possibly suggesting the direct effect of the biogenic structures associated with this bivalve: *Mya* siphon activity (its withdrawal and extension) occurs at a depth of 5 cm and organic matter is presumably concentrated here, a dense growth of microorganisms is recorded here and this might attract nematodes. Hence, the effect of *Mya* on nematodes may be very localized, with a lack of response in the surrounding sediment.

The nematode species numbers in both bivalve treatments and in the defaunated control were reduced compared to those from the field community. *D.* aff*. setosus*, *T. marinus*, *L. papilliger* and *V. viscosia* did not survive in the experimental control, indicating sensitivity of these species to the absence of a bioturbator. *D*. *cephalata*, *V*. *viscosia* and *C.* cf. *honestus* were not recorded in the *Cerastoderma* and *Mya* microcosms, and these species were probably additionally sensitive to the absence of an active and mobile bioturbator such as *Hediste*.

The response of nematodes to the presence of *H. diversicolor* in our experiment was distinct and significant. The most striking observation was that nematode assemblages in all treatments with *Hediste*, both in monoculture and with *Cerastoderma* or *Mya*, were usually more similar than replicates within any other single treatment, including the control (Fig. 5). The nematode assemblages in all *Hediste* treatments also differed significantly from those recorded in other treatments. In addition, the change in nematode community structure in the presence of *Hediste* was uniform, regardless of the presence or absence, and identity, of any accompanying species.

The filter- and/or surface deposit-feeding *H. diversicolor* builds a system of U-shaped burrows with numerous branches creating galleries below the sediment surface (Kristensen and Kostka 2005 and refs therein). Sediment inhabited by several worms is therefore characterized by a high concentration of galleries creating a dense network that affects the surrounding sediment, probably in a relatively uniform way compared to the effect of bivalves. The effect of this network of burrows and associated modifications in the surrounding sediment is a change in nematode community structure and composition.

The dissimilarity of assemblages recorded in the polychaete treatments (H, HC, HM) compared to other treatments was largely caused by changes in abundance of *A. elongatus*, *A. thalassophygas, H. disjuncta* and *C.* cf. *honestus*. The changes in each of these species may represent different effects of *Hediste* on the nematode community: The reduced occurrence of *A. thalassophygas* in *Hediste* treatments compared to the defaunated control but similar to that observed in the field may result from competition between the polychaete and the nematode for food resources. Also, physical disturbance of the upper sediment layers by *H. diversicolor* foraging activity may result in increased mortality of *A. thalassophygas* usually concentrated at the sediment surface. Tita et al. (2000) proposed that this foraging strategy, described as ‘sweeping and ploughing’ the sediment surface, may cause destruction of nematode pathways, thus reducing food interception and therefore reducing nematode feeding success. Sediment surface disturbance might have been also the reason for the observed reduction in the abundance of *A. thalassophygas* in *Cerastoderma* treatments compared to the control. It is worth noting that in *Mya*-only microcosms where the sediment surface was not disturbed by the bivalve, there was no change in *A. thalassophygas* abundance compared to the control.

The habitat created deeper in the sediment due to the presence of *Hediste* supported another large nematode, *A. elongatus.* This nematode tended to occupy deeper sediment depths even in the absence of the macrofauna (the control treatment) and the addition of *Hediste* clearly facilitated its survival and its penetration into deeper sediment, probably in response to newly available food sources produced by the polychaete burrowing activity [this is probably also the case for other non-selective deposit feeders such as *A. viviparum* and *D. zeelandicus* (Fig. 4, Table 3)].

Microalgal food transported into the sediment column by *Hediste* burrow irrigation probably attracted the diatom-feeding *Calomicrolaimus honestus*, which penetrated the sediment to a depth of 15 cm. Interestingly, occurrence of *C. honestus* in the field was limited just to deeper sediment layers (5–10, 10–15 cm). *H. disjuncta*, on the other hand, was limited to the sediment surface in the field and increased in microcosms with the bivalves (C, M, CM) probably owing to the increased microbial food resource associated with faecal pellets. Warwick et al. (1986) and Austen et al. (1998) proposed that the food provided by bacteria growing on freshly deposited faeces and pseudofaeces produced by bivalves may attract certain nematode species and can be responsible for differences in their fine scale distribution. *H. disjuncta* feeds predominantly on bacteria, but also on algae, diatoms and ciliates (Moens and Vincx 1997). There is, therefore, the possibility that in our experiment *H. disjuncta* was attracted to bacteria and/or food sources provided by bacteria and diatoms growing on bivalve faeces and pseudofaeces deposited on the sediment surface.

The redistribution of some nematode species over depth layers in the *Hediste* treatments was in contrast to their restriction to the sediment surface in bivalve microcosms and was likely to have been responsible for the observed differences in nematode community structure between treatments. The presence of the polychaete also seemed to maintain more diverse nematode communities than those recorded in control and bivalve treatments. We can hypothesize that the more uniform vertical distribution of nematodes in treatments with *Hediste* probably decreased competition processes within the community owing to its increased diversity (Joint et al. 1982; Pinto et al. 2006). In addition, extension of the habitat might have led to niche separation and consequently the reduced dominance.

Changes in nematode community composition may also result in changes in diversity. For instance, the reduced dominance of *A. thalassophygas* in *Hediste* treatments most likely allowed other species to co-exist with it, thus leading to the enhanced diversity recorded. On the other hand, *Hediste* might have facilitated the survival of more nematode species, thus leading to the reduction in *A. thalassophygas* dominance.

Conversely, in the study by Austen et al. (1998), meiofauna diversity did not differ between contrasting macrofaunal disturbance types. These findings might be explained by the functional differences between the macrofaunal species they studied: here, we found that *Mya* and *Cerastoderma* treatments did not result in differences in nematode diversity, probably owing to their relatively low activity levels and limited effect on sediment processes. Thus, it is quite likely that similarly the functional differences between the macrofaunal species studied by Austen et al. (1998) were not sufficiently contrasting to record different responses in nematode diversity. Furthermore, these authors studied subtidal nematode community from sandy mud sediment. Meiofauna from this type of sediment is limited to the sediment surface (nematodes were sampled to a depth of 5 cm only) and might be less sensitive or more tolerant to the presence of bioturbator and associated sediment modifications and changes in sediment characteristics.

The response of the nematode community appeared to be independent of the intensity of disturbance in our experiment. The change in nematode community structure in the presence of *Hediste* was uniform, regardless of its density and/or biomass, which were different between monocultures and two-species treatments. This agrees with the field observations of Pinto et al. (2006), who found that although the deposit-feeding polychaete *Laeonereis acuta* increased the vertical penetration of nematodes, as does *Hediste* in our study, different densities of this polychaete had no effect on nematode community structure. Further, Austen et al. (1998) showed that the species-specific response of nematode assemblages to macrofaunal activity was much more dependant on the type of activity than its quantity (expressed as bivalve density).

The predominant effect of *Hediste* on interstitial characteristics and processes rates, as observed in multi-species mixtures (*Hediste diversicolor*, *Cerastoderma edule* and *Corophium volutator*) by Mermillod-Blondin et al. (2005), seems also to be mirrored in nematode communities inhabiting the interstitial environment. In two-species treatments, *Hediste* had a predominant effect on nematodes, indicating that not the species number but their identity determines the response of nematode community. Our study, however, focuses on two-species mixtures and the next step should be to include more species, representing more functional traits, to test the effect of these multi-species assemblages on nematode communities.

Our study highlights the importance of using multiple approaches to understand ecosystem functioning, in the context of ecological and economic impacts of biodiversity, and emphasizes the need for a more holistic approach when studying benthic systems. When studying the infauna, we should be careful not to limit the environmental characteristics measured to physical sedimentary properties as is frequently the case, but consider a wider range of parameters, both biotic and abiotic, that may influence, for example, nematode occurrence and community structure.

## References

[CR1] Anderson MJ, Gorley RN, Clarke KL (2008). PERMANOVA + for PRIMER: guide to software and statistical methods.

[CR2] Austen MC, Widdicombe S (1998). Experimental evidence of effects of the heart urchin *Brissopsis lyrifera* on associated subtidal meiobenthic nematode communities. J Exp Mar Biol Ecol.

[CR3] Austen MC, Widdicombe S, Villano-Pitacco N (1998). Effects of biological disturbance on diversity and structure of meiobenthic nematode communities. Mar Ecol Prog Ser.

[CR4] Biles C, Solan M, Isaksson I, Paterson D, Emes C, Raffaelli D (2003). Flow modifies the effect of biodiversity on ecosystem functioning: an in situ study of estuarine sediments. J Exp Biol Ecol.

[CR5] Blott SJ, Pye K (2001). Gradistat: a grain size distribution and statistics package for the analysis of unconsolidated sediments. Earth Surf Proc Land.

[CR6] Braeckman U, Provoost P, Gribsholt B, Van Gansbeke D, Middelburg JJ, Soetaert K, Vincx M, Vanaverbeke J (2010). Role of macrofauna functional traits and density in biogeochemical fluxes and bioturbation. Mar Ecol Prog Ser.

[CR7] Braeckman U, Van Colen C, Soetaert K, Vincx M, Vanaverbeke J (2011). Contrasting macrobenthic activities differentially affect nematode density and diversity in a shallow subtidal marine sediment. Mar Ecol Prog Ser.

[CR8] Braeckman U, Provoost P, Moens T, Soetaert K, Middelburg JJ, Vincx M, Vanaverbeke J (2011). Biological *vs.* physical mixing effects on benthic food web dynamics. PLoS ONE.

[CR9] Christensen B, Vedel A, Kristensen E (2000). Carbon and nitrogen fluxes in sediment inhabited by suspension-feeding (*Nereis diversicolor*) and non-suspension-feeding (*N. virens*) polychaetes. Mar Ecol Prog Ser.

[CR10] Emmerson MC, Raffaelli DG (2000). Detecting the effects of diversity on measures of ecosystem function: experimental design, null models and empirical observations. Oikos.

[CR11] Emmerson MC, Solan M, Emes C, Paterson DM, Raffaelli DG (2001). Consistent patterns and the idiosyncratic effects of biodiversity in marine ecosystems. Nature.

[CR12] Ertman SC, Jumars PA (1988). Effects of bivalve siphon currents on the settlement of inert particles and larvae. J Mar Res.

[CR13] Forster S, Zettler ML (2004). The capacity of the filter-feeding bivalve *Mya arenaria* L. to affect water transport in sandy beds. Mar Biol.

[CR14] Grasshoff K, Erhardt M, Kremling K (1983). Methods of sea water analysis.

[CR15] Hansen K, King GM, Kristensen E (1996). Impact of the soft-shell clam *Mya arenaria* on sulfate reduction in an intertidal sediment. Aquat Microb Ecol.

[CR16] Hedman JE, Gunnarsson JS, Samuelsson G, Gilbert F (2011). Particle reworking and solute transport by the sediment-living polychaetes *Marenzelleria neglecta* and *Hediste diversicolor*. J Exp Mar Biol Ecol.

[CR17] Heip C, Vincx M, Smol N, Vranken G (1982). The systematics and ecology of free-living marine nematodes. Helminthol Abstr Ser B.

[CR18] Heip C, Vincx M, Vranken G (1985). The ecology of marine nematodes. Oceanogr Mar Biol Ann Rev.

[CR19] HELCOM (1988) Guidelines for the Baltic monitoring programme for the third stage, physical and chemical determinants in sea water. In: Baltic Sea environment proceedings. No. 27B, p 60

[CR20] Henriksen K, Rasmussen MB, Jensen A (1983). Effect of bioturbation on microbial nitrogen transformations in the sediment and fluxes of ammonium and nitrate to the overlying water. Ecol Bull.

[CR21] Ieno EN, Solan M, Batty P, Pierce GJ (2006). How diversity affects ecosystem functioning: roles of infaunal species richness, identity and density in the marine benthos. Mar Ecol Prog Ser.

[CR22] Joint R, Gee JM, Warwick RM (1982). Determination of fine scale vertical distribution of microbes and meiofauna in an intertidal sediment. Mar Biol.

[CR23] Karlson K, Bonsdorff E, Rosenberg R (2007). The impact of benthic macrofauna for nutrient fluxes from Baltic Sea sediments. Ambio.

[CR24] Kennedy AD (1993). Minimal predation upon meiofauna by endobenthic macrofauna in the Exe Estuary, south west England. Mar Biol.

[CR25] Kristensen E (2001) Impact of polychaetes (*Nereis* spp. and *Arenicola marina*) on carbon biogeochemistry in coastal marine sediments. Geochem Trans 2–92 10.1186/1467-4866-2-92 PMC1475601 16759424

[CR26] Kristensen K, Hansen K (1999). Transport of carbon dioxide and ammonium in bioturbated (*Nereis diversicolor*) coastal, marine sediments. Biogeochemistry.

[CR27] Kristensen E, Kostka JE (2005). Macrofaunal burrows and irrigation in marine sediments: microbiological and biogeochemical interactions. In: Kristensen E, Haese RR, Kostka JE (eds) Interactions between macro- and microorganisms in marine sediments. Coast Estuar Stud.

[CR28] Majdi N, Mialet B, Boyer S, Tackx M, Leflaive J, Bouleˆtreau S, Ten-Hage L, Julien F, Fernandez R, Buffan-Dubau E (2012) The relationship between epilithic biofilm stability and its associated meiofauna under two patterns of flood disturbance. Freshw Sci 31(1):38–50

[CR29] Mermillod-Blondin F, Francois-Carcaillet F, Rosenberg R (2005). Biodiversity of benthic invertebrates and organic matter processing in shallow marine sediments: an experimental study. J Exp Mar Biol Ecol.

[CR30] Moens T, Vincx M (1997). Observations on the feeding ecology of estuarine nematodes. J Mar Biol Assoc UK.

[CR31] Norling K, Rosenberg R, Hulth S, Gremare A, Bonsdorf E (2007). Importance of functional biodiversity and species-specific traits on benthic fauna for ecosystem functions in marine sediment. Mar Ecol Prog Ser.

[CR32] Olafsson E (2003). Do macrofauna structure meiofauna assemblages in marine soft-bottoms? A review of experimental studies. Vie Milieu.

[CR33] Olafsson E, Elmgren R (1991). Effects of biological disturbance by benthic amphipods *Monoporeia affinis* on meiobenthic community structure: a laboratory approach. Mar Ecol Prog Ser.

[CR34] Olafsson E, Elmgren R, Papakosta O (1993). Effects of the deposit-feeding benthic bivalve *Macoma balthica* on meiobenthos. Oecologia.

[CR35] Pinto TK, Austen MC, Bemvenuti CE (2006). Effects of macro-infauna sediment disturbance on nematode vertical distribution. J Mar Biol Assoc UK.

[CR36] Platt HM, Warwick RM, Price JH, Irvine DEG, Farnham WF (1980). The significance of free living nematodes to the littoral ecosystem. The shore environment.

[CR37] Platt HM, Warwick RM (1983) Freeliving marine nematodes. Part I. British Enoplids. In: Kermack DM, Barnes RSK (eds) Synopses of the British fauna (New Series) No. 28. Cambridge University Press, Cambridge, p 307

[CR38] Platt HM, Warwick RM (1988) Freeliving marine nematodes. Part II. British Chromadorids. In: Kermack DM, Barnes RSK (eds) Synopses of the British fauna (New Series), No. 38. E.J. Brill/Dr. W. Backhuys, Leiden, p 501

[CR39] Raffaelli D, Emmerson M, Solan M, Biles C, Paterson D (2003). Biodiversity and ecosystem processes in shallow coastal waters: an experimental approach. J Sea Res.

[CR40] Reise K (1979). Moderate predation on meiofauna by the macrobenthos of the Wadden Sea. Helgolander Meeresun.

[CR41] Reise K (1983). Biotic enrichment of intertidal sediments by experimental aggregates of the deposit-feeding bivalve *Macoma balthica*. Mar Ecol Prog Ser.

[CR42] Ricciardi A, Bourget E (1998). Weight-to-weight conversion factors for marine benthic macroinvertebrates. Mar Ecol Prog Ser.

[CR43] Schratzberger M, Warwick RM (1999). Differential effects of various types of disturbances on the structure of nematode assemblages: an experimental approach. Mar Ecol Prog Ser.

[CR44] Schratzberger M, Warwick RM (1999). Impact of predation and sediment disturbance by *Carcinus maenas* (L.) on free-living nematode community structure. J Exp Mar Biol Ecol.

[CR45] Tita G, Desrosiers G, Vincx M, Clément M (2000). Predation and sediment disturbance effects of the intertidal polychaete *Nereis virens* (Sars) on associated meiofaunal assemblages. J Exp Mar Biol Ecol.

[CR46] Ullberg J, Olafsson E (2003). Effects of biological disturbance by *Monoporeia affinis* (Amphipoda) small-scale migration of marine nematodes in low-energy soft sediments. Mar Biol.

[CR47] Van Colen C, Rossi F, Montserrat F, Andersson MGI, Gribsholt B (2012). Organism-sediment interactions govern post-hypoxia recovery of ecosystem functioning. PLoS ONE.

[CR48] Vincx M (1996) Meiofauna in marine and freshwater sediments. In: Hall GS (ed) Methods for the examination of organismal diversity in soil and sediments. CAB International

[CR49] Volkenborn N, Hedtkamp SIC, van Beusekom JEE, Reise K (2007). Effects of bioturbation and bioirrigation by lugworms (*Arenicola marina*) on physical and chemical sediment properties and implications for intertidal habitat succession. Estuar Coast Shelf Sci.

[CR50] Waldbusser GG, Marinelli RL, Whitlatch RB, Visscher PT (2004). The effects of infaunal biodiversity on biogeochemistry of coastal marine sediments. Limnol Oceanogr.

[CR51] Warwick RM, Gee JM, Berge JA, Ambrose WJ (1986). Effects of the feeding activity of the polychaete S*treblosoma bairdi* on meiofaunal abundance and community structure. Sarsia.

[CR52] Warwick RM, McEvoy AJ, Thrush SF (1997). The influence of *Atrina zelandica* Gray on meiobenthic nematode diversity and community structure. J Exp Mar Biol Ecol.

[CR53] Warwick RM, Platt HM, Somerfield PJ (1998) Free-living marine nematodes. Part III. Monhysterids. In: Barnes RSK, Crothers JH (eds) Synopses of the British fauna (New Series) No. 53. Field Studies Council, Shrewsbury, p 296

[CR54] Wetzel MA, Leuchs H, Koop JHE (2005). Preservation effects on the wet weight, dry weight, and Ash-free dry weight biomass estimates of four common estuarine macro-invertebrates: no defence between ethanol and formalin. Helgoland Mar Res.

[CR55] Wieser W (1953). Beziehungen zwischen Mundhhlengstalt, Ernahrungsweise und Vorkommen bei Freilebenden marinen Nematoden. Ark Zool.

